# Multi-organ dysfunction and outcomes in pregnancy associated COVID-19 infection – descriptive review of pathological findings

**DOI:** 10.1186/s12884-023-06240-x

**Published:** 2024-01-09

**Authors:** Ya.G. Turdybekova, I.L. Kopobayeva, A. A. Turmukhambetova, Y.K. Kamyshanskiy

**Affiliations:** Karaganda Medical University, Karaganda, Kazakhstan

**Keywords:** Maternal death associated with COVID-19, Multiple organ microvasculitis

## Abstract

**Objective(s):**

Comparative clinical and morphological characterization of lesions of the vascular and nervous system in cases of maternal death associated with COVID-19.

**Study design:**

The study included autopsy in 12 cases of maternal death with a positive intravital result for SARS-CoV-2 by reverse transcription polymerase chain reaction. For histopathology, tissue samples were taken from the internal organs of each patient. Pieces of organs were fixed and stained according to the standard protocol. The relative number of microvessels with vasculitis and fibers of the peripheral nervous system with infiltration by immune cells was studied. All morphological changes were classified depending on the severity of the damage.

**Results:**

The average age of patients with a fatal outcome was 35 ± 4.4 years. Time to death after onset of symptoms averaged 16 ± 4.4 days. Dystrophic lesions (necrosis and apoptosis) of the villous and extravillous trophoblast and decidual tissue were observed in the studied placentas. Histopathological signs of mild and severe lesions of the peripheral nervous system in the organs of the gastrointestinal tract were detected in 2 (16.7%) and 10 (83.3%) cases, respectively, in the myocardium in 4 (33.3%) and 8 (66.7%) cases. Histopathological signs of severe damage to the microvascular bed in the organs of the gastrointestinal tract were registered in 9 (75%) cases.

**Conclusion(s):**

The main clinical feature of this cohort was that death occurred in a long-term period, in most cases with a negative PCR. The histopathological pattern was a non-acute injury with an immune component of the microvascular bed and the autonomic nervous system with predominant damage to the myocardium and intestines.

**What does this study add to the clinical work:**

This study makes it possible to even better study the immunopathological profile in organs and tissues in pregnant women with a fatal outcome when affected by a viral infection, in particular Covid-19. This knowledge can be used when humanity encounters other viral pandemics in the future.

## Introduction

COVID-19 (SARS-CoV-2) has been the cause of a pandemic and death worldwide for 2.5 years. COVID-19 has often been mild or asymptomatic in pregnant women, but severe and fatal cases have also been reported. Documentation of the histopathological features of the disease caused by SARS-CoV-2 is sparse in reported cases of maternal death. The pathogenesis of COVID-19 is not known: the proposed thanatogenesis involves immune-mediated mechanisms or direct effects of SARS-CoV-2 on tissues.

Pregnant women form a special group of patients with specific immunological and physiological changes [1–3]. Weakening of cell-mediated immune responses makes a pregnant woman more susceptible to infections caused by intracellular pathogens such as viruses [4]. Physiological changes during pregnancy have a significant impact on the respiratory, cardiovascular, and coagulation systems, which may have different effects on the progression of COVID-19 disease.

The impact of SARS-CoV-2 on pregnancy remains to be established, as the pathophysiological mechanisms are unclear, and the morphological patterns of damage to organs and organ systems are heterogeneous without signs of tissue and organ specificity. Previously, it was shown [5–8], that damage associated with COVID-19 is accompanied by damage to the vascular and peripheral nervous systems, histopathologically manifested by systemic multiple organ microvasculitis [9, 10].

The objective of our work was a comparative clinical and morphological characterization of lesions of the vascular and nervous system in cases of maternal death associated with COVID-19.

## Methods

### Study design

The study included 12 maternal deaths from COVID-19 with a positive intravital nasopharyngeal swab for SARS-CoV-2 using reverse transcription polymerase chain reaction that occurred in 2020–2021. An autopsy was performed in the pathoanatomical department. Lifetime testing for SARS-CoV-2 was performed by licensed medical laboratories using polymerase chain reaction (PCR) nasal and throat swab samples. The written informed consent of the next of kin was obtained for the autopsy. Clinical information and laboratory data were obtained from patients’ electronic health records.

### Autopsy procedure and tissue processing

The autopsy took from 3 to 22 h (average 11 h) after death.

External examination, autopsy, and tissue sampling of all patients were performed in accordance with the latest recommendations for safety precautions in suspected or confirmed cases of COVID-19, including the use of masks, protective suits, and cut-resistant gloves. The autopsy was performed in accordance with the autopsy protocol developed in our organization in accordance with published guidelines [11–13]. In all cases, a complete autopsy was performed with a study of the brain, all organs of the chest and abdominal cavity.

For histopathology, tissue samples were taken from the internal organs of each patient, from which, after macroscopic evaluation, the most representative areas were selected. Photographs were taken and representative sections of all organs and clearly visible lesions were presented.

We fixed organ pieces in neutral buffered formalin (10%) and stained with hematoxylin & eosin according to the standard protocol. Two licensed pathologists fulfilled the histological examination of autopsy material.

### Morphological study

The relative number of microvessels with vasculitis and fibers of the peripheral nervous system with infiltration by immune cells was assessed in histological sections.

All morphological changes were classified depending on the severity of the damage. The absence of morphological changes was classified as *absent* (no infiltration by immune cells), *low* (< 30% of vessels/nerve fibers) and *high* (> 30% of vessels/nerve fibers).

### Descriptive statistics

Continuous data with a normal distribution were expressed as an average value ± standard deviation (SD), whereas continuous data with a nonnormal distribution were represented as a median (interquartile range). Categorical data were represented in a frequency (as a percentage).

## Results

### General cohort characteristics and the results of clinical and laboratory examination

The average age of patients with a fatal outcome was 35 ± 4.4 years. The most common initial symptoms were dyspnea, fever, and cough. Additional respiratory pathogens were isolated from two patients. The median time from symptom onset to hospitalization was 4 days (2.75–5.25). All patients were intubated. The median time from symptoms onset to intubation was 9 days (8.0–10.75). Time to death after symptoms onset ranged from 12 to 26 days, with the average value of 16 ± 4.4 days. In all cases, computed tomography showed bilateral multifocal diffuse ground-glass opacities to consolidation, with a median area of involvement of 52% (range 25–88%). Three patients had premature detachment of a normally located placenta. Characteristics of the cohort, clinical symptoms, and data from laboratory and instrumental studies are presented in Table 1.

**Table 1:** Characteristics of the disease development from the onset of symptoms to death in pregnant women and puerperas with confirmed SARS-CoV-2 infection

№	Age	Concomitant extragenital pathology	Initial symptoms	Time from symptom onset to admission – to intubation – to death, days	Volume of lung tissue damage	Additional respiratory pathogens	Pathology of pregnancy and childbirth
1	32	-	Dyspnea, occasional dry cough, fever, chills, sweating, weakness	4–8 – 18	65%	-	PDNLP
2	37	Obesity of the II^nd^ degree	Dry cough, fever, headaches	1–8 – 14	40%	-	-
3	24	Type I diabetes mellitus, chronic arterial hypertension, primary hypothyroidism, severe anemia	Dyspnea, severe general weakness, diarrhea, nausea	8–8 – 12	85%	-	SP, impairment of blood flow of 1 A degree
4	35	Gestational diabetes mellitus, chronic arterial hypertension, obesity of the III^rd^ degree	Dry cough, nasal congestion, sore throat, nausea, vomiting, diarrhea	7–7 – 16	25%	Yes	-
5	40	-	Weakness, sore throat	4–10 – 12	35%	-	-
6	32	-	Fever, dyspnea, weakness, shortness of breath, nausea, diarrhea, vomiting	6–10 – 14	30%	-	-
7	35	-	Cough, fever, shortness of breath, weakness	2–17 – 26	76%	Yes	PDNLP
8	32	-	Cough, shortness of breath, fever, weakness	3–7 – 17	36%	Yes	-
9	36	Chronic pyelonephritis	Sore throat, sweating, fever	2–10 – 12	76%		-
10	36	Moderate anemia	Weakness, fever, cough	5–8 – 21	56%	-	PDNLP
11	38	Reactive arthritis, chronic arterial hypertension of the II^nd^ degree	Dry cough, weakness, joint pain, fever	3–13 – 13	28%	-	-
12	40	-	Fever, cough with phlegm, weakness	4–15 – 20	80%	Yes	-

PDNLP – premature detachment of a normally located placenta, SP – severe preeclampsia

Delivery was performed by caesarean section in all cases, in 3 cases – up to 37 weeks. Antenatal fetal death occurred in two patients, in one patient the child died on the 5th day after birth (respiratory distress syndrome, critically low body weight). The average weight of full-term newborns was 3108 g. The duration of neonatal hospitalization varied from 4 to 55 days. Pneumonia/sepsis of the newborn was observed in 5 cases. Perinatal outcomes for maternal deaths following confirmed SARS-CoV-2 infection are presented in Table 2.

**Table 2:** Perinatal outcomes in maternal deaths after confirmed SARS-CoV-2 infection

Gestational period	Antenatal fetal death / neonatal death	Method of delivery	Weight at birth	Neonatal hospitalization, days	Apgar	Pneumonia / sepsis / respiratory distress syndrome
23 + 5	yes	C-section	650	-	-	-
34 + 2	-	C-section	2790	21	1–2	yes
31 + 6	-	C-section	2595	15	7–7	no
38	-	C-section	4060	5	7–8	no
37 + 6	-	C-section	2890	13	1–2	yes
36	-	C-section	3290	5	7–8	no
37	-	C-section	3200	4	7–8	no
30 + 4	-	C-section	1800	55	7–7	yes
23 + 5	yes	C-section	360	-	-	-
37	-	C-section	3200	-	7–8	no
25 + 3	yes	C-section	700	5	2–4	yes
36 + 1	-	C-section	2845	21	5–7	yes

C-section – cesarean section

Severe edematous lungs were revealed at autopsy in all the dead, the average weight for the right lung was 1439 g, for the left lung – 950 g. The lung tissue was heavy and hard, uneven bluish-red color with diffuse hemorrhages. The volume of pleural fluid varied greatly and ranged from 0 to 350 ml per pleural cavity. Histopathological examination of the respiratory system demonstrated a pattern of diffuse alveolar injury in 100% of patients, as evidenced by the presence of intraalveolar fibrin, hyaline membranes, or organizing connective tissue in the alveolar septa. Patterns of acute (exudative) and organizing (proliferative) diffuse alveolar lesions were identified in 10 (83.3%) of 12 patients with diffuse alveolar lesions.

Alveolar hemorrhagic syndrome with diffuse alveolar damage was identified in 8 (66.7%) of 12 patients. All patients demonstrated heterogeneous patterns of acute and subacute injury with marked perivascular lymphocytic infiltration. Foci of acute purulent inflammation were also determined: focal bronchial or bronchoalveolar inflammation was revealed in 3 patients. Focal pulmonary microthrombi were detected in 10 (83.3%) patients.

Gastrointestinal pathological findings in the liver showed mild periportal lymphocytic inflammation, focal centrilobular necrosis consistent with hypoperfusion injury. Nonspecific acute histopathological changes were detected in the large and small intestines, including mucosal edema, erosions and focal necrosis. All patients demonstrated heterogeneous patterns of microvasculature damage of varying severity, including infiltration of the vessel wall by immune cells.

Acute and subacute ischemic changes were revealed in the brain, including destructive changes in nerve cells such as swelling, vacuolization, karyolysis and perivascular glial hyperplasia. All patients except №3 and №4 had vasculitis of varying severity.

Non-specific acute histopathological changes and damage to the microvasculature and peripheral nervous system were found in the myocardium, including infiltration by immune cells and chronic damage (perivascular sclerosis and lipomatosis, focal hypertrophy of cardiomyocytes). An organized thrombus was diagnosed in the heart in patient №7.

Signs suggestive of acute tubular injury, including extensive vacuolization of the tubular epithelium, have been identified in the kidneys.

Severe dystrophic changes in the myometrium, myonecrosis, microthrombi and vasculitis in the uterus were detected in 11 cases. The uterus was not found at autopsy in 1 case (patient №8), as it was removed during a caesarean section due to atony.

Dystrophic lesions (necrosis and apoptosis) of the villous and extravillous trophoblast and decidual tissue were observed in the studied placentas: lymphocytic vasculitis, perivascular lymphocytic clutches and vasculitis in the area of the placental bed with a high degree of endothelial damage (pronounced edema and swelling, desquamation of the endothelium from the basal plate, apoptosis, necrosis), thrombi of the intervillous space. In 1 case (patient №1), acute intervillositis with obliteration of the intervillous space was detected. Histological findings for individual patients are presented in Table 3.

**Table 3:** Histopathological changes in the organs of deceased patients

№	Lungs	Brain	Intestine	Heart	Liver	Spleen	Kidneys	Uterus	Placenta
1	DAD, proliferative type; interstitial infiltrates of lymphocytes and histiocytes, acute bacterial pneumonia, alveolar/capillary megakaryocytes, vasculitis, microthrombi	Edema, perivascular hemorrhage, karyolysis of neurons, vasculitis, microthrombi	Edema, erosion, necrosis, microvasculitis	Perivascular sclerosis, focal necrosis	Cholestasis, mild steatosis, centrilobular necrosis, lymphocytic infiltrates, microvasculitis	Lymphoid depletion, reduction of lymphoid follicles	Partial necronephrosis, vacuolation of the tubular epithelium, microthrombi	Myonecrosis, microthrombi	Placental abruption, acute intervillusitis, necrosis of the villous trophoblast, thrombi of the interstitial space, meconium in amniotic membranes, severe MVM
2	DAD, mixed type; interstitial infiltrates of lymphocytes, acute bacterial pneumonia, hemorrhages, alveolar megakaryocytes, microthrombi	Edema, neuronal karyolysis, microthrombi, vasculitis	Edema, necrosis, microvasculitis	Hypertrophy of cardiomyocytes, vasculitis	Severe steatosis, microvasculitis	Lymphoid depletion, reduction of lymphoid follicles	Partial necronephrosis, vacuolation of the tubular epithelium	Myonecrosis, microthrombi	Necrosis of the villous trophoblast, thrombosis of the interstitial space, severe MVM
3	DAD, mixed type; infiltrates of lymphocytes and histiocytes, alveolar/capillary megakaryocytes, vasculitis	Edema, microthrombi	Edema, erosion, vasculitis	Perivascular sclerosis	Mild steatosis, vasculitis	No significant changes	Arterionephrosclerosis, lymphocytic infiltration, focal segmental glomerulosclerosis, microthrombi	Edema, vasculitis	Mild FVM
4	DAD, mixed type; alveolar/capillary megakaryocytes, vasculitis	Edema, neuron karyolysis	Edema, erosion, vasculitis	Hypertrophy of cardiomyocytes, focal necrosis of cardiomyocytes	Mild steatosis, vasculitis	No significant changes	Mild arterionephrosclerosis, lymphocytic infiltration, microthrombi	Myonecrosis, vasculitis	Severe MVM, mild FVM
5	DAD, mixed type; interstitial infiltrates of lymphocytes and histiocytes, alveolar/capillary megakaryocytes, vasculitis, microthrombi	Edema, swelling, karyolysis of neurons, microthrombi, vasculitis	Edema, necrosis, erosion, vasculitis	Lymphocytic infiltrate, microthrombi	Moderate steatosis, lymphocytic infiltrates, cholestasis	Lymphoid depletion, reduction of lymphoid follicles	Mild arterionephrosclerosis, lymphocytic infiltration, microthrombi	Myonecrosis, vasculitis, microthrombi	Necrosis of the extracellular trophoblast, thrombosis of the intercellular space, severe MVM
6	DAD, mixed type; alveolar/capillary megakaryocytes, vasculitis, microthrombi	Edema, karyolysis of neurons, microthrombi, vasculitis	Edema, dystrophic changes	Focal necrosis of cardiomyocytes	Moderate steatosis	No significant changes	No significant changes	Vasculitis, microthrombi	Necrosis of the villous trophoblast, severe MVM
7	DAD, proliferative type; interstitial infiltrates of lymphocytes and histiocytes, alveolar/capillary megakaryocytes, vasculitis, microthrombi	Edema, swelling, karyolysis of neurons, microthrombi, vasculitis	Edema, necrosis, vasculitis	Subendocardial thrombi, focal necrosis, lymphocytic infiltration, microthrombi, vasculitis	Mild steatosis, focal centrilobular necrosis, vasculitis	Lymphoid depletion, reduction of lymphoid follicles	General necronephrosis, microthrombs	Myonecrosis, vasculitis, microthrombi	Placental abruption
8	DAD, mixed type; vasculitis, microthrombi	Edema, vasculitis	Edema, dystrophic changes, necrosis	No significant changes	Mild steatosis, cholestasis	No significant changes	Subtotal necronephrosis, microvasculitis	-	-
9	DAD, mixed type; vasculitis, microthrombi	Edema, microthrombi, vasculitis	Edema, hemorrhages	No significant changes	Mild steatosis, necrosis, reactive hepatitis	Reduction of lymphoid follicles	Partial necronephrosis, vacuolation of the tubular epithelium	Thrombosis of the uterine arteries	Mild chorioamnionitis without fetal inflammatory response
10	DAD, proliferative type, interstitial infiltrates of lymphocytes and histiocytes, acute bacterial pneumonia, alveolar megakaryocytes, vasculitis, microthrombi	Karyolysis of neurons, vasculitis	Vasculitis	Лимфоцитарный инфильтрат	Reactive hepatitis, vasculitis	Reduction of lymphoid follicles, microvasculitis	Total necronephrosis, lymphocytic infiltration, microvasculitis	Vasculitis, microthrombi	Placental abruption, severe MVM
11	Common thrombosis of the pulmonary arteries and microvessels of both lungs	Karyolysis of neurons, vasculitis	Vasculitis	No significant changes	No significant changes	Reduction of lymphoid follicles, vasculitis, hemophagocytosis	No significant changes	Vasculitis, microthrombi	Necrosis of the villous trophoblast, mild MVM, mild FVM
12	Interstitial fibrosis, vasculitis, microthrombi	Edema, neuronal karyolysis, vasculitis	Edema, hemorrhages, vasculitis	No significant changes	Mild steatosis	Reduction of lymphoid follicles	Subtotal necronephrosis	No significant changes	Severe MVM, mild FVM

DAD – diffuse alveolar damage, MVM – maternal vascular malperfusion, FVM – fetal vascular malperfusion,

### Histopathological pattern of microvascular lesions

Histopathological patterns of mild and severe lesions of the microvascular bed were found in the organs of the respiratory system in 3(25.0%) and 9(75.0%) cases.

Vasculitis of microvessels was not detected in the organs of the gastrointestinal tract or occupied less than 30% of the vessels in 3(25.0%) cases. Immune cells affected more than 30% of microvasculature vessels in 9(75%) cases.

Histopathological signs of damage to the microvasculature in the organs of the central nervous system were not detected in 5(41.6%) cases, the histopathological pattern of mild microvascular damage was detected in 7(58.3%) cases.

Microvascular vasculitis in the myocardium was not detected in 4(33.3%) cases, microvascular vasculitis occupied less than 30% of the vessels in 5(41.69%) cases. Histopathological pattern of severe microvascular damage was found in 3(25.01%) cases.

### Histopathological pattern of lesions in the peripheral nervous system

Histopathological signs of mild and severe damage to the peripheral nervous system in the organs of the gastrointestinal tract were detected in 2(16.7%) and 10(83.3%) cases.

Fibers of the peripheral nervous system with infiltration by immune cells, damaging less than 30% of the fibers, were found in the myocardium in 4(33.3%) cases, fibers of the peripheral nervous system with infiltration by immune cells, damaging more than 30% of the fibers, in 8(66, 6%) cases.

## Discussion

### Principal findings

The spectrum of pathology in people dying from COVID-19 is just beginning to emerge [14–16]. We present a case series of 12 maternal deaths following confirmed SARS-CoV-2 infection. Our results demonstrate an important role in the mechanism of thanatogenesis in severe SARS-CoV-2 infection in capillary and neurotrophic insufficiency of internal organs and provide evidence-indicating damage to microvessels and the peripheral nervous system by immune cells.

An important histopathological finding is the fact that in all the presented cases, non-specific damage to microvessels (vasculitis by immune cells) and the peripheral nervous system (perineural edema with intranerval infiltration by immune cells) with selective damage to the myocardium and intestines was found. Moderate or severe perivascular mononuclear chronic inflammatory cell aggregates were observed in almost all cases. Severe injuries were associated with a longer course of the disease, in some cases with multiple negative PCR results in the long-term period. This morphological picture may be a substrate for progressive multiple organ failure, which partly explains the mechanisms of thanatogenesis in «non-recovering» patients. Longer follow-up may be important, as most of our patients have died within days or weeks of first symptoms and often in the postpartum period.

The direct cause of death of patients was multiple organ failure with subsequent negative PCR in 9(75%) cases. In a morphological study, we found that a fatal outcome after a confirmed infection with SARS-CoV-2 was often associated with diffuse microvascular and neurotrophic insufficiency, the morphological substrate of which was the infiltration of microvascular walls and fibers of the peripheral nervous system by immune cells with the predominant involvement of myocardial and intestinal tissues. The selectivity of damage remains debatable and requires further research. In the long term, this process can lead to severe damage with an autoimmune component.

### Clinical implications

The histopathological picture of the uteroplacental complex was characterized by diffuse damage to the placental link (necrosis and apoptosis of the villous and extravillous trophoblast, thrombi in the intervillous space, vasculitis). In the study cohort, a high incidence of PDNLP was observed – 3(25.0%) cases. It is not excluded that an additional factor that increased the risk of PDNLP in this case could be apoptosis of the chorionic and decidual tissue due to SARS CoV-2 infection. It is likely that apoptosis is associated with direct viral (cytopathic) or secondary (hypoxic) damage. The pathogenesis of the damage is most likely as follows: apoptosis of villous and extravillous trophoblast cells, combined with necrosis due to hypoxic damage caused by systemic endotheliitis, promotes the production of prostaglandins and the release of cytokines, which increases uterine contractions and leads to placental abruption. This assumption requires the further research.

### Results in the context of what is known

As in previously published scientific studies [17, 18], the histopathological observation in our series of patients who died from COVID-19 was diffuse alveolar lung disease with a heterogeneous mosaic pattern in the acute (exudative) and organizational (proliferative) phases. This lesion was also observed in a patient who died in 4 days after hospitalization, suggesting that lung injury in patients with COVID-19 may be subclinical and clinically relevant for the identification and management of patients with diffuse alveolar injury.

We observed a difference in the average age of the mother in our case series compared with other cohorts [19], the average age of the deceased was higher in our cohort.

The clinical significance of this difference is unknown. The presence in 3 cases of comorbidities that increase the population risk (diabetes mellitus, gestational diabetes mellitus, chronic arterial hypertension, hypothyroidism) and preeclampsia is an additional factor that should be taken into account when interpreting the results of this study. Similarly, we consider it unlikely that the quality of obstetric care provided could have contributed to poor outcomes.

All 12 maternal deaths associated with COVID-19 infection occurred in women who had never received a dose of the COVID-19 vaccine. Our case series is consistent with studies indicating that severe SARS-CoV-2 infection increases the risk of maternal death in unvaccinated women [20–23].

### Research implications

The findings require further clinical and morphological studies of the immunopathological process as a pathophysiological process of inflammatory damage to the microvascular bed and peripheral nervous system with an immune component. The important clinical significance of this fact lies in the need to develop new strategies for managing such cases in confirmed SARS-CoV-2 infection.

### Strengths and limitations

Our study has limitations. The number of cases studied was limited to 12 patients, further studies will show whether the relatively consistent results of this study of a small number of patients are consistent with larger cohorts.

## Conclusions

The results of our studies have shown that the histology of microvessels and fibers of the peripheral nervous system demonstrates pathological changes (infiltration by immune cells) in cases of maternal death associated with COVID-19. The main clinical feature of this cohort was that death occurred in a long-term period, in most cases with a negative PCR. The main clinical and morphological complex was microvascular and neurotrophic insufficiency of internal organs (clinically - multiple organ failure). The histopathological pattern was a non-acute injury with an immune component of the microvascular bed and the autonomic nervous system with predominant damage to the myocardium and intestines.

## Data Availability

All data generated or analysed during this study are included in this published article (and its supplementary information files-Tables).
